# Development of a CRISPR/Cas9-Based Tool for Gene Deletion in *Issatchenkia orientalis*

**DOI:** 10.1128/mSphere.00345-19

**Published:** 2019-06-26

**Authors:** Vinh G. Tran, Mingfeng Cao, Zia Fatma, Xiaofei Song, Huimin Zhao

**Affiliations:** a Department of Chemical and Biomolecular Engineering, Carl R. Woese Institute for Genomic Biology, University of Illinois at Urbana-Champaign, Urbana, Illinois, USA; b Department of Microbiology, Nankai University, Tianjin, China; c Department of Chemistry, University of Illinois at Urbana-Champaign, Urbana, Illinois, USA; d Department of Biochemistry, University of Illinois at Urbana-Champaign, Urbana, Illinois, USA; e Department of Bioengineering, University of Illinois at Urbana-Champaign, Urbana, Illinois, USA; Carnegie Mellon University

**Keywords:** CRISPR/Cas9, *Issatchenkia orientalis*, genome editing, metabolic engineering, synthetic biology

## Abstract

The nonconventional yeast Issatchenkia orientalis has emerged as a potential platform microorganism for production of organic acids due to its ability to grow robustly under highly acidic conditions. However, lack of efficient genetic tools remains a major bottleneck in metabolic engineering of this organism. Here we report that the autonomously replicating sequence (ARS) from Saccharomyces cerevisiae (ScARS) was functional for plasmid replication in *I. orientalis*, and the resulting episomal plasmid enabled efficient genome editing by the CRISPR/Cas9 system. The optimized CRISPR/Cas9-based system employed a fusion *RPR1*′*-tRNA* promoter for single guide RNA (sgRNA) expression and could attain greater than 97% gene disruption efficiency for various gene targets. Additionally, we demonstrated multiplexed gene deletion with disruption efficiencies of 90% and 47% for double gene and triple gene knockouts, respectively. This genome editing tool can be used for rapid strain development and metabolic engineering of this organism for production of biofuels and chemicals.

**IMPORTANCE** Microbial production of fuels and chemicals from renewable and readily available biomass is a sustainable and economically attractive alternative to petroleum-based production. Because of its unusual tolerance to highly acidic conditions, *I. orientalis* is a promising potential candidate for the manufacture of valued organic acids. Nevertheless, reliable and efficient genetic engineering tools in *I. orientalis* are limited. The results outlined in this paper describe a stable episomal ARS-containing plasmid and the first CRISPR/Cas9-based system for gene disruptions in *I. orientalis*, paving the way for applying genome engineering and metabolic engineering strategies and tools in this microorganism for production of fuels and chemicals.

## Abstract (precis)

Microbial production of fuels and chemicals from renewable and readily available biomass is a sustainable and economically attractive alternative to petroleum-based production. Because of its unusual tolerance to highly acidic conditions, *I. orientalis* is a promising potential candidate for the manufacture of valued organic acids. Nevertheless, reliable and efficient genetic engineering tools in *I. orientalis* are limited. The results outlined in this paper describe a stable episomal ARS-containing plasmid and the first CRISPR/Cas9-based system for gene disruptions in *I. orientalis*, paving the way for applying genome engineering and metabolic engineering strategies and tools in this microorganism for production of fuels and chemicals.

## INTRODUCTION

Owing to its extraordinary tolerance to multiple stresses, including extremely low-pH conditions, Issatchenkia orientalis is a promising platform microorganism for the production of organic acids. It was previously used in ethanol fermentation at pH 2 (1) and engineered to produce succinic acid (2) and lactic acid (3). However, the tools for genetic engineering in *I. orientalis* are very limited, which significantly prohibits extensive metabolic engineering efforts. Stable episomal plasmids and efficient genome editing tools are the two foundational technologies for genetic engineering (4), which *I. orientalis* is currently lacking. Episomal plasmids allow rapid genetic manipulations and render microorganisms genetically tractable. The core functional element of episomal plasmids is an autonomously replicating sequence (ARS). ARS is a DNA replication starting point, similar to the origin of replication in bacteria, and it directs the replication of the genomic DNA and episomal plasmid (5, 6). For Saccharomyces cerevisiae, episomal plasmids include centromere (CEN)-based low-copy-number plasmids and 2μ-based high-copy-number plasmids (4). In contrast, there is no available stable episomal plasmid for *I. orientalis*, and even a functional ARS has not been identified.

In addition to the lack of episomal plasmids, *I. orientalis* is devoid of precise genome editing tools, in particular CRISPR/Cas (clustered regularly interspaced short palindromic repeats and CRISPR-associated proteins). CRISPR/Cas9 is a powerful tool for rapid genome engineering in which a single guide RNA (sgRNA) containing a spacer sequence complementary to the targeted DNA sequence guides Cas9, a DNA endonuclease enzyme, to a genomic target (7, 8). Upon binding, Cas9 creates a DNA double-strand break (DSB). DNA repair mechanisms, homologous recombination (HR) or nonhomologous end joining (NHEJ), can be exploited to introduce gene insertions and deletions. CRISPR/Cas9 has been successfully implemented with high editing efficiencies in various species, such as Escherichia coli, S. cerevisiae, and mammalian cells (9–12). A CRISPR/Cas9-based tool for *I. orientalis*, however, has not yet been developed.

In this study, we report a functional ARS for plasmid replication and the first CRISPR/Cas9-based system for targeted and markerless gene disruption in *I. orientalis*. A variety of promoters for sgRNA expression were characterized, and knockouts of several genes were performed with efficiencies greater than 97%. We also demonstrated efficient multiplexed genome editing by disrupting *ADE2* and *TRP1*; *ADE2* and *HIS3*; and *ADE2*, *HIS3*, and *SDH2* with 72.8%, 89.9%, and 46.7% disruption efficiencies, respectively. Our optimized CRISPR/Cas9 system represents a powerful tool for comprehensive metabolic engineering of *I. orientalis* to produce biofuels and chemicals.

## RESULTS

### Replicable plasmid endowed by S. cerevisiae ARS.

**FIG 1:**
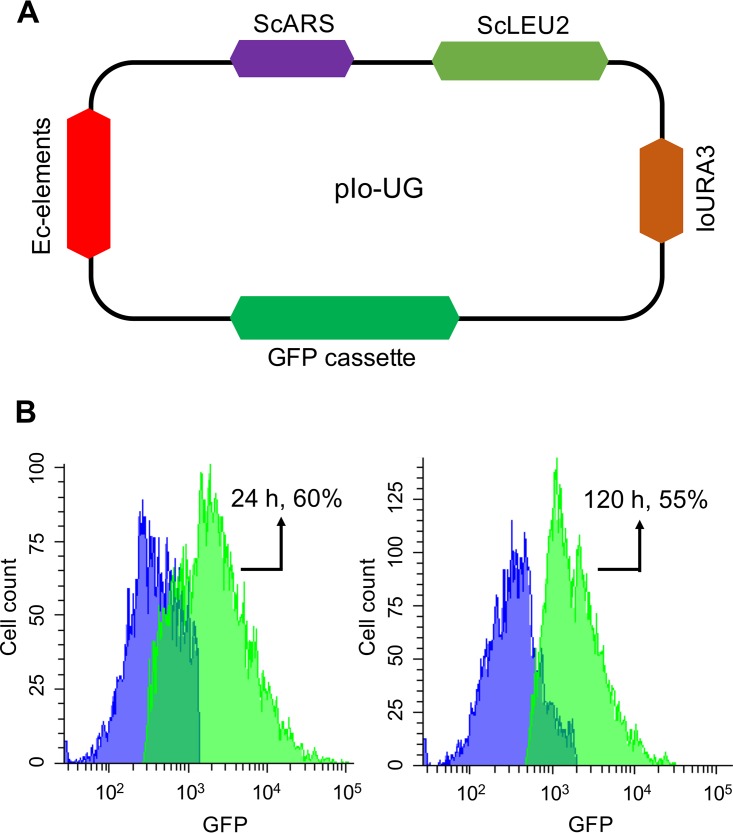
Design and construction of an episomal plasmid, pIo-UG. (A) pIo-UG map containing *I. orientalis URA3* selection marker, GFP expression cassette, E. coli elements (Ec-elements), S. cerevisiae ARS (ScARS), and *LEU2* selection marker (ScLEU2). (B) The GFP expression peaks at 24 h and 120 h measured by flow cytometry.

Based on a recent study on *I. orientalis* population genomics, there was a high similarity of the genome sequences between *I. orientalis* and S. cerevisiae (13). Hence, we hypothesized that the well-characterized ARS from S. cerevisiae (ScARS) may be functional in *I. orientalis*. To test this hypothesis, we used the DNA assembler method (14) to construct a plasmid (pIo-UG), which was derived from pRS415, containing the *I. orientalis* uracil auxotrophic selection marker (*IoURA3*), ScARS, S. cerevisiae LEU2 (ScLEU2), and a green fluorescent protein (GFP) gene as a reporter (Fig. 1A). Approximately 1000 colonies were obtained with 500 ng pIo-UG by heat shock transformation (see Fig. S1A in the supplemental material), and around 55% of the cells cultured in liquid media could express the GFP at a symmetric peak for at least 5 days (Fig. 1B). However, we did not observe any colony growing for the control plasmid pIo-control (without ScARS) on an SC-URA (SC-uracil) plate (Fig. S1B). Then, plasmid was extracted from *I. orientalis* cultured for 120 h and transformed to E. coli. We were still able to see E. coli colonies (Fig. S1C), and plasmid extracted from E. coli was confirmed to be the original plasmid pIo-UG by restriction digestion (Fig. S1D). Thus, the ScARS-containing plasmid could be maintained in *I. orientalis*. It was also reported that CEN sequence could greatly improve plasmid stability in S. cerevisiae, and the plasmid with CEN showed >80% GFP expression (4). We tested the functionality of the centromere from S. cerevisiae (ScCEN) in *I. orientalis*. However, no improvement was obtained by the addition of ScCEN to pIo-UG (Fig. S1E). Since CEN is essential to direct precise DNA segregation, isolation of a functional CEN is the goal of our future work.

10.1128/mSphere.00345-19.2 FIG S1 DNA transformations and GFP expression. (A) *I. orientalis* transformation by heat shock with 500 ng of pIo-UG. (B) *I. orientalis* transformation by heat shock with 500 ng of pIo-control (without ScARS). (C) E. coli transformation by electroporation with plasmid DNA extracted from 24-h and 120-h *I. orientalis* cultures. (D) Digestion confirmation of plasmid extracted from S1C by XbaI+NotI (two bands, 8 kb and 1.7 kb; ladder, New England BioLabs [NEB] 1 kb Plus DNA ladder). (E) Profiles of GFP expression at 24 h by ScARS and ScARS/CEN plasmids in *I. orientalis*. Download FIG S1, TIF file, 2.4 MB. Copyright © 2019 Tran et al. 2019 Tran et al. This content is distributed under the terms of the Creative Commons Attribution 4.0 International license.

### CRISPR/Cas9 system in *I. orientalis*.

Having obtained a functional ARS, we next sought to design a CRISPR/Cas9-based tool for *I. orientalis*. An efficient CRISPR/Cas9 system requires functional Cas9 and sgRNA expressions. Cas9 expression can be achieved by using a constitutive RNA polymerase (RNAP) II promoter. On the other hand, sgRNA expression typically requires an RNAP III promoter because of the mRNA processing associated with RNAP II, such as 5′ end capping and 3′ end polyadenylation (15). Should RNAP II promoter be used for sgRNA expression, the sgRNA needs to be flanked with self-cleaving delta virus ribozyme sequences (16). These ribozymes can execute cleavage on both ends of sgRNA and release the mature sgRNA without posttranscriptional modifications.

In yeasts, genes transcribed by the RNAP III promoter include all the *tRNA* genes, *SNR6* (U6 spliceosomal RNA), *SNR52* (C/D box small nucleolar RNA), *RPR1* (RNA component of nuclear RNase P), *SCR1* (RNA subunit of the signal recognition particle), and 5S rRNA (17). These promoters have been used to drive sgRNA expression in other yeast species. For example, *SNR52* and 5S rRNA were used for sgRNA expression in S. cerevisiae and Aspergillus niger, respectively (10, 18). tRNA by itself can act as a promoter, and fusion of tRNA with other promoters, such as the hybrid promoter *SCR1*′*-*tRNA^Gly^ in Yarrowia lipolytica, can excise the sgRNA from the primary transcript by the tRNA maturation processing (19). Hence, it is possible to use these RNAP III promoters to express sgRNA in *I. orientalis*. The partial sequence of *RPR1* in *I. orientalis* ATCC 6258 was previously identified (20), and there was a homolog in *I. orientalis* SD108. 5S rRNA in *I. orientalis* was identified by BLAST search against the 5S rRNA of S. cerevisiae S288C. These two genes serve as the starting point for sgRNA expression in our CRISPR/Cas9-based system.

**FIG 2:**
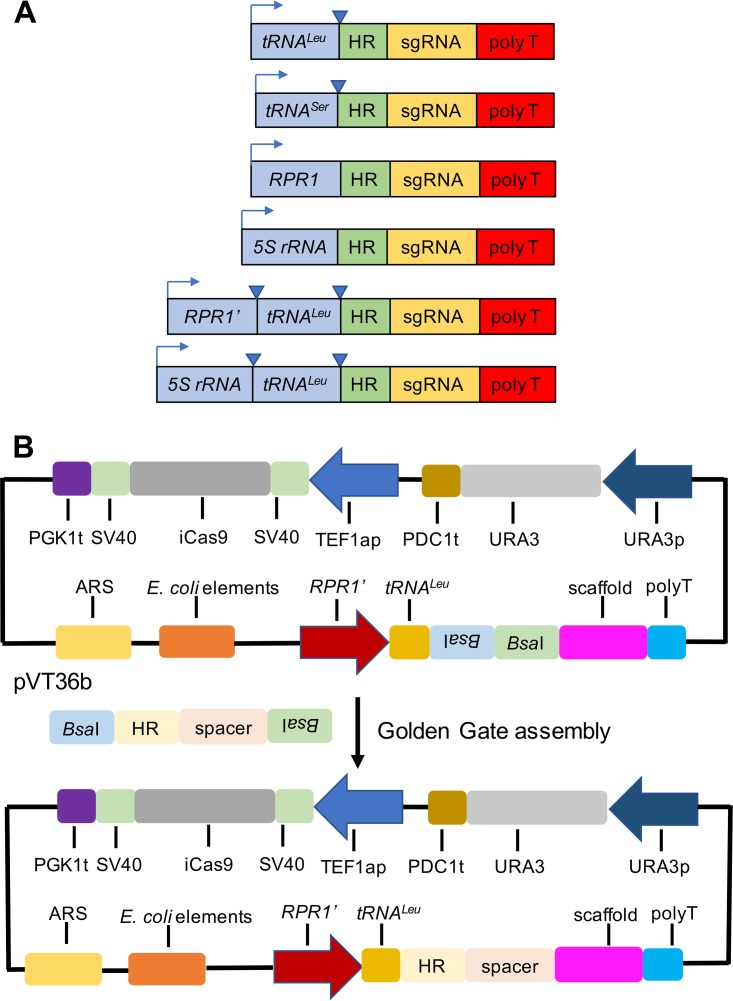
CRISPR/Cas9 system. (A) Constructs of various promoters for sgRNA expression. Triangles indicate tRNA cleavage site. (B) Scheme showing the design of CRISPR/Cas9 plasmid with *RPR1*′-tRNA^Leu^ as the promoter for sgRNA expression (pVT36b) and the Golden Gate cloning method to assemble gBlock containing HR donor and spacer into the plasmid.

A series of promoters was evaluated, including a leucine tRNA (tRNA^Leu^), a serine tRNA (tRNA^Ser^), 5S rRNA, *RPR1*, and fusions of 5S rRNA and *RPR1*′ with tRNA^Leu^ (Fig. 2A). The *RPR1* promoter contains 250 bp upstream of *RPR1* partial sequence, while the *RPR1*′ promoter contains 250 bp upstream of *RPR1* and first 120 bp of *RPR1*. The promoter elements of *RPR1* can be located upstream or internal to the mature product (17). However, the exact promoter elements of *RPR1* from *I. orientalis* are unknown. Therefore, we tested two different *RPR1* promoters. The Cas9 used in this system is iCas9, which is short for improved Cas9 and was shown to yield higher disruption efficiency in S. cerevisiae than the wild-type Cas9 (21). iCas9 was tagged with simian virus 40 (SV40) nuclear localization sequences at both N and C termini and driven by a strong constitutive promoter, *TEF1ap*.

**FIG 3:**
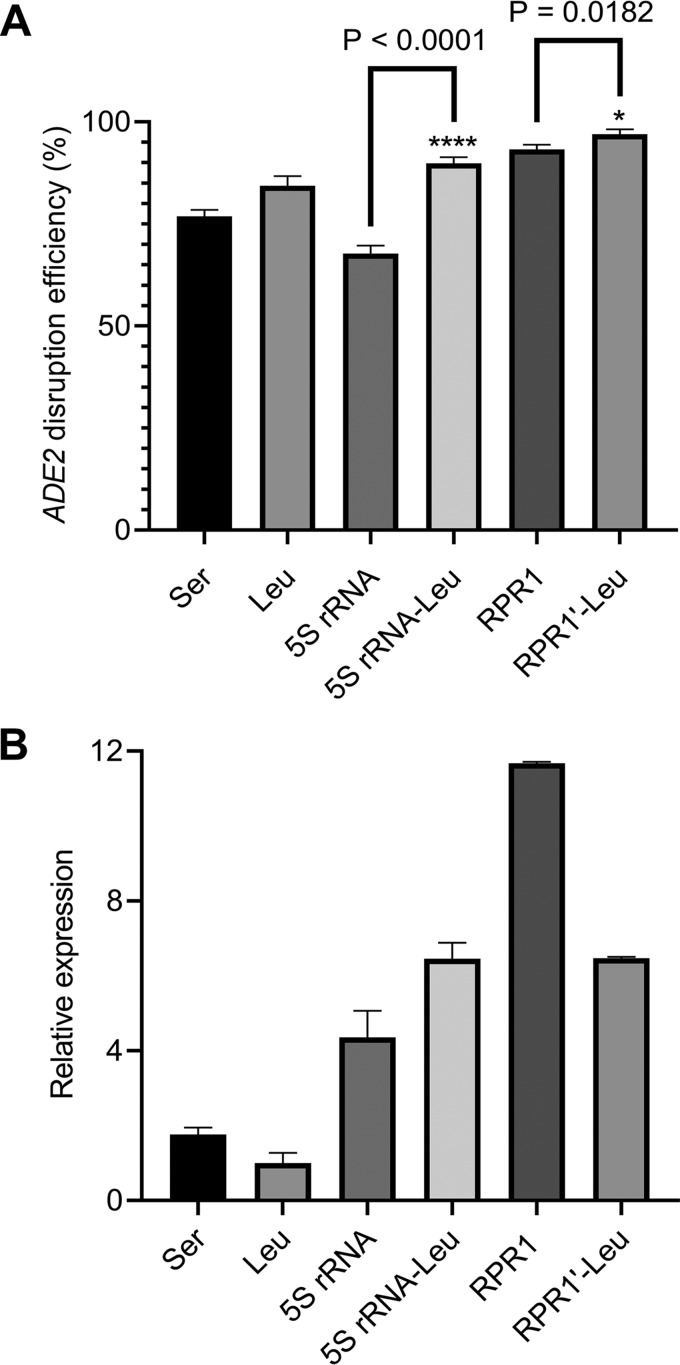
*ADE2* disruption. (A) *ADE2* knockout efficiencies using different promoters for sgRNA expression. All asterisks indicate statistical difference (*P < *0.05) calculated using a two-tailed type II Student *t* test. Error represents standard deviation for biological triplicates. (B) qPCR analysis of sgRNA expression levels for different promoters. Data shown are sgRNA level normalized to the sgRNA level generated by the tRNA^Leu^ promoter. *alg9* was used as the reference gene. Error bars represent standard deviations for biological triplicates.

As a proof of concept, we targeted the *ADE2* gene because the *ADE2* nonsense mutant shows a conspicuous red phenotype, which could be screened for quickly (21). Initially, we did not know whether HR or NHEJ was the dominant repairing mechanism in *I. orientalis*. First, we evaluated the HR mechanism by including an HR disruption in the plasmid. A gBlock containing an HR donor and spacer for *ADE2* disruption was cloned into CRISPR/Cas9 plasmid using the Golden Gate assembly method (22), and in the resulting plasmid, the HR donor was fused to the 5′ end of sgRNA (Fig. 2B). The HR donor contained an 8-bp deletion in the middle, and two 50-bp-homology arms flanked both sides of the centered 8-bp deletion (21). The 8-bp deletion included the protospacer adjacent motif (PAM) sequence and the last 3 bp of the spacer. If HR was the primary mode of DNA DSB repair, the defined 8 bp would be removed from the genome, resulting in a frameshift mutation. Various CRISPR/Cas9 constructs with different promoters for sgRNA expression were transformed into *I. orientalis*, and all transformants were plated on SC-URA plates. Colonies were then screened for the red and white phenotypes. For all cases, growing the cells for a prolonged period of time in liquid SC-URA after transformation was not necessary to observe *ADE2* disruption. The highest *ADE2* disruption efficiency of 97.0% ± 1.0% (number of red colonies/number of total colonies: 200/209, 320/326, 300/309) was attained with *RPR1*′*-*tRNA^Leu^ promoter (Fig. 3A). The *RPR1* and 5S RNA-tRNA^Leu^ promoters also produced high-efficiency *ADE2* disruptions, 93.3% ± 0.9% (140/150, 128/139, 100/106) and 89.8% ± 1.2% (200/227, 190/210, 200/220), respectively. The tRNA^Leu^, tRNA^Ser^, and 5S rRNA promoters resulted in lower disruption efficiencies, 84.4% ± 1.9% (250/306, 280/327, 320/373), 76.9% ± 1.3% (141/187, 115/150, 110/140), and 67.8% ± 1.6% (4/6, 7/10, 4/6), respectively. To confirm *ADE2* disruption, 4 red colonies were randomly picked and sent for sequencing, and DNA sequencing analysis showed deletion of the defined 8 bp for all colonies (Fig. S2A), indicating that our CRISPR/Cas9 system was functional for gene disruption and *I. orientalis* could utilize HR to repair DNA DSB. Next, to test if *I. orientalis* was capable of accomplishing NHEJ-based DNA repair, only the spacer was cloned into the CRISPR/Cas9 plasmid with *RPR1*′-tRNA^Leu^ promoter. Following transformation of *I. orientalis* with the plasmid, less than 10 transformants appeared, and they all retained the wild-type white color (Fig. S2B). In addition, plasmid with *RPR1*′-tRNA^Leu^ promoter lacking both HR donor and sgRNA was transformed into *I. orientalis*, and the number of colonies appearing was greater than 10^5^ (Fig. S2C). Taken together, these data suggested that HR is the main DNA repair mechanism.

10.1128/mSphere.00345-19.3 FIG S2 *ADE2* disruption. (A) DNA sequencing analysis for *ADE2* disruption. (B) Transformation of plasmid with *RPR1*′-tRNA^Leu^ promoter with sgRNA targeting *ADE2*. HR donor was not included in the plasmid. (C) Transformation of plasmid with *RPR1*′-tRNA^Leu^ promoter lacking both HR donor and sgRNA. Download FIG S2, TIF file, 2.6 MB. Copyright © 2019 Tran et al. 2019 Tran et al. This content is distributed under the terms of the Creative Commons Attribution 4.0 International license.

To determine whether sgRNA expression levels correlate with *ADE2* disruption efficiencies, qPCR was employed to quantify the transcription levels of sgRNAs (Fig. 3B). Transcript levels produced from tRNA^Leu^, tRNA^Ser^, and 5S rRNA promoters were lower than those produced from other promoters, which might explain the lower *ADE2* knockout efficiencies. *RPR1* promoter produced approximately 2-fold more sgRNA than the *RPR1*′-tRNA^Leu^ promoter, yet the *ADE2* disruption efficiency by the *RPR1* promoter was not as high as that by the *RPR1*′-tRNA^Leu^ promoter.

To validate if the *RPR1*′-tRNA^Leu^ promoter would consistently produce higher knockout efficiency, despite the *RPR1* promoter producing the largest amount of sgRNA, several additional genes were chosen for disruptions using both *RPR1* and *RPR1*′-tRNA^Leu^ promoters to express sgRNA. The disruption efficiencies between these promoters were then compared. *LEU2*, *HIS3*, and *TRP1* are essential for yeasts to synthesize their own leucine, histidine, and tryptophan, respectively. Successful disruptions of these genes will also result in mutants with *leu2*, *his3*, or *trp1* auxotrophy. Random colonies were picked and screened for knockout using liquids SC-URA and SC minus appropriate auxotrophic compound. A disruption efficiency of 100% (10/10, 10/10) was obtained for *LEU2* and *TRP1* knockouts for both *RPR1* and *RPR1*′-tRNA^Leu^ promoters (Table 1 and Fig. S3). However, the efficiency of *HIS3* disruption by *RPR1* promoter, 90% ± 10% (8/10, 10/10), was lower than that by *RPR1*′-tRNA^Leu^ promoter, 100% (10/10, 10/10). Five colonies from each disruption using *RPR1*′-tRNA^Leu^ promoter were sent for sequencing, and they were all confirmed to be disrupted at the defined target site. Since *RPR1*′-tRNA^Leu^ promoter generally resulted in higher disruption efficiencies, it was chosen as the final promoter for sgRNA expression for subsequent knockouts.

**TABLE 1:** Comparison of disruption efficiencies between *RPR1* and *RPR1*′-tRNA^Leu^ promoters

Gene	Disruption efficiency by promoter (%)	
	*RPR1*	*RPR1*′-tRNA^Leu^
*LEU2*	100	100
*HIS3*	90 ± 10	100
*TRP1*	100	100

10.1128/mSphere.00345-19.4 FIG S3 Disruptions of auxotrophic genes, *LEU2*, *HIS3*, and *TRP1*, using both *RPR1* and *RPR1*′-tRNA^Leu^ promoters. (A) *LEU2* disruption with *RPR1* promoter. (B) *HIS3* disruption with *RPR1* promoter. (C) *TRP1* disruption with *RPR1* promoter. (D) *LEU2* disruption with *RPR1*′-tRNA^Leu^ promoter. (E) *HIS3* disruption with *RPR1*′-tRNA^Leu^ promoter. (F) *TRP1* disruption with *RPR1*′-tRNA^Leu^ promoter. Only one biological duplicate of each knockout is shown. Download FIG S3, TIF file, 2.7 MB. Copyright © 2019 Tran et al. 2019 Tran et al. This content is distributed under the terms of the Creative Commons Attribution 4.0 International license.

**FIG 4:**
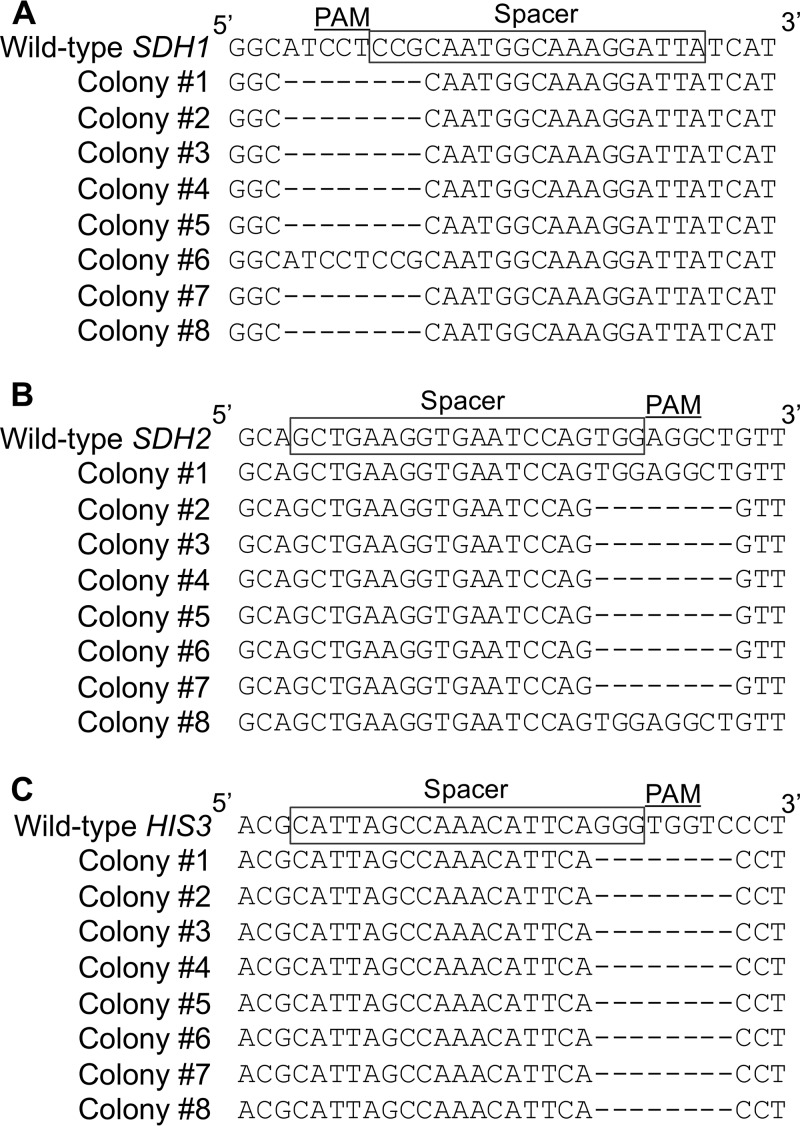
DNA sequencing analysis. (A) *SDH1* disruption. (B) *SDH2* disruption in triple-gene disruption. (C) *HIS3* disruption in triple-gene disruption.

To also demonstrate that our CRISPR/Cas9 system can be used to knock out genes whose disruptions do not result in visible phenotypic change, *SDH1* and *SDH2*, encoding subunits 1 and 2 of succinate dehydrogenase, respectively, were chosen for single-gene and triple-gene disruption. In the *SDH1* knockout, 8 colonies were randomly picked and screened by PCR and DNA sequencing, and 7 out of 8 colonies were shown to be disrupted at the chosen target site (Fig. 4A).

### Multiplexed gene disruption using CRISPR/Cas9.

**FIG 5:**
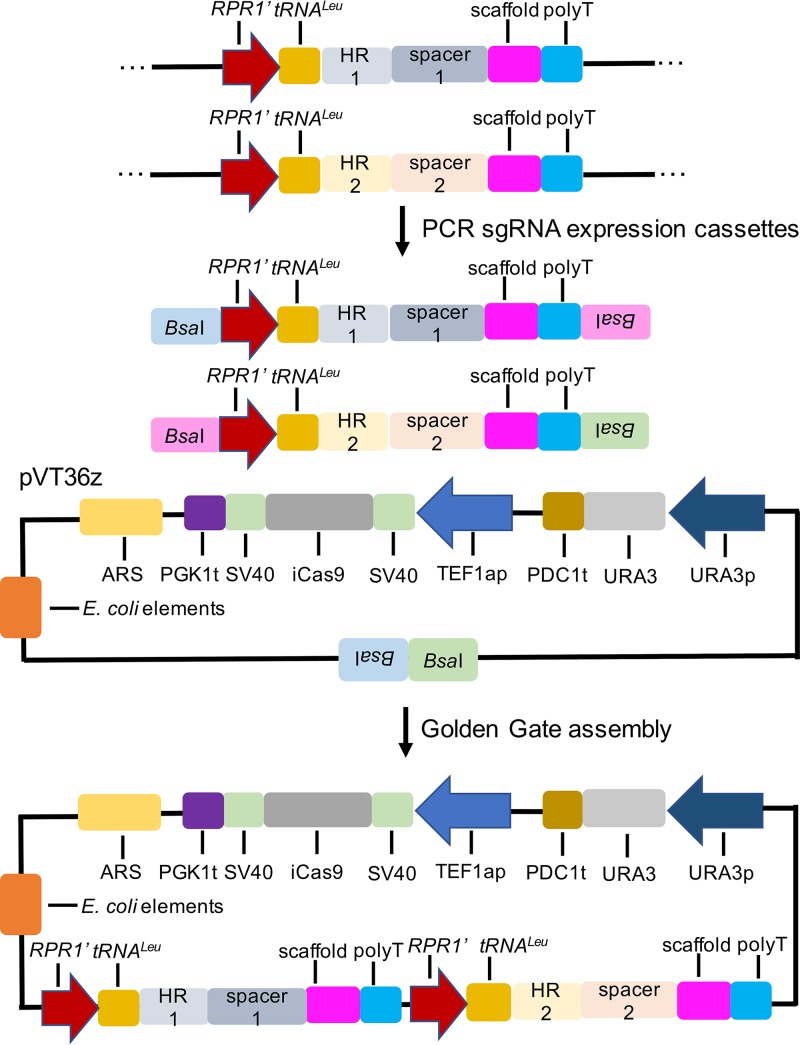
Scheme showing the assembly of CRISPR/Cas9 plasmid for multiplexed gene deletions. gBlock containing HR donor and spacer for each target site was first assembled into pVT36b. sgRNA expression cassettes were then PCR amplified, and the Golden Gate assembly method was used to clone the cassettes into pVT36z.

In addition to single-gene knockouts, we examined our CRISPR/Cas9 system for multiplexed gene deletions. Cassettes for sgRNA expressions were separately PCR amplified and sequentially cloned into CRISPR/Cas9 plasmid (Fig. 5). *ADE2* and *TRP1* and *ADE2* and *HIS3* were selected for double gene knockouts. The multiplexed CRISPR plasmid was transformed into *I. orientalis*, and all transformants were plated on an SC-URA plate. Independently of the *TRP1* or *HIS3* knockout efficiency, the overall *ADE2* editing efficiencies were determined to be 89.5% ± 0.6% (113/127, 100/111) and 95.9% ± 0.7% (200/210, 200/207) for *ADE2*/*TRP1* and *ADE2*/*HIS3* double-gene knockouts, respectively. For each knockout, 8 randomly picked red colonies were then screened for loss of *TRP1* or *HIS3* function using SC-URA and SC lacking appropriate auxotrophic nutrients. *TRP1* and *HIS3* disruption efficiencies were determined to be 81.3% ± 6.3% (6/8, 7/8) and 93.8% ± 6.3% (8/8, 7/8), respectively (Fig. S4). All things considered, in double-gene knockouts, *ADE2* and *TRP1* and *ADE2* and *HIS3* were deleted with efficiencies estimated to be 72.8% ± 6.0% and 89.9% ± 5.3%, respectively (Table 2).

**TABLE 2:** Multiplexed gene disruption efficiencies

Genes	Disruption efficiency (%)
*ADE2* and *TRP1*	72.8 ± 6.0
*ADE2* and *HIS3*	89.9 ± 5.3
*ADE2*, *HIS3*, and *SDH2*	46.7

10.1128/mSphere.00345-19.5 FIG S4 Double-gene disruptions. (A) *ADE2* and *TRP1* disruption. (B) *ADE2* and *HIS3* disruption. Only one biological duplicate of each knockout is shown. Download FIG S4, TIF file, 2.2 MB. Copyright © 2019 Tran et al. 2019 Tran et al. This content is distributed under the terms of the Creative Commons Attribution 4.0 International license.

Last, we also tested whether our CRISPR/Cas9 system could disrupt 3 genes simultaneously in a single transformation. *ADE2*, *HIS3*, and *SDH2* were selected as target genes. The overall disruption efficiency of *ADE2* was estimated to be 62.3% (114/183). Eight red colonies were then randomly picked and screened for *HIS3* and *SDH2* knockouts by sequencing. Among these 8 colonies, 6 of them had both *HIS3* and *SDH2* disrupted (Fig. 4B and C). Thus, *ADE2*, *HIS3*, and *SDH2* were simultaneously disrupted with an efficiency of 46.7% (Table 2).

## DISCUSSION

The yeast *I. orientalis* is renowned for its unique ability to tolerate several stresses, including extremely low pH (2). Thus, it is a microorganism that can potentially be engineered to produce organic acids. Nevertheless, engineering endeavors in this species will be hindered until efficient and consistent synthetic biology tools are established. In this study, we determined that the ScARS allowed stable plasmid replication and maintenance in *I. orientalis*. Around 55% of the cells grown for 5 days in liquid culture still carried the plasmid and expressed GFP. This episomal plasmid will be a useful tool for rapid genetics and allow expression of biological parts without permanently integrating the parts into the chromosome.

CRISPR/Cas9 is an emerging genome engineering tool that has revolutionized biotechnology research (7). Developing a CRISPR/Cas9 system in *I. orientalis* posed some technical challenges. RNAP III promoters in many nonconventional yeasts, including *I. orientalis*, are not well characterized (23). We were unable to identify the potential sequences for the endogenous *SNR52* and *SCR1*. It might be possible that these promoters could achieve even greater gene disruption efficiency than our *RPR1*′-tRNA promoter. Furthermore. *I. orientalis* is diploid, and diploid and polyploid industrial yeasts can be particularly challenging to engineer due to the difficulty and necessity of disrupting multiple copies of one gene (24). Despite these challenges, we have developed a CRISPR/Cas9-based tool that could achieve high gene disruption efficiency in *I. orientalis*. We examined several promoters for sgRNA expression and found that the *RPR1* and *RPR1*′-tRNA^Leu^ promoters exhibited high *ADE2* disruption rates. The *RPR1*′-tRNA^Leu^ promoter increased *ADE2* knockout efficiency slightly but significantly compared to *RPR1* promoter (*P* = 0.0182) (Fig. 2B). Furthermore, qPCR results indicated that the *RPR1* promoter produced more sgRNA transcripts than the *RPR1*′-tRNA^Leu^ promoter, but efficiencies of gene knockout by the *RPR1*′-tRNA^Leu^ promoter were generally higher for various single-gene targets. Nevertheless, it is not unusual that the promoter with the highest sgRNA expression does not result in the highest disruption efficiency. It was reported that in Y. lipolytica, the *SNR52*′-tRNA^Gly^ promoter produced the largest amount of sgRNA, around 2-fold more sgRNA than the *SCR1*′-tRNA^Gly^ promoter (19). However, the highest *PEX10* disruption efficiency was achieved by using the *SCR1*′-tRNA^Gly^ promoter.

Our CRISPR/Cas9 system also could achieve high efficiency in multiplexed gene deletions, 90% and 47% for double-gene and triple-gene disruptions, respectively. In haploid S. cerevisiae, triple-gene disruption could be achieved with an efficiency of 100% (21). In the industrial triploid S. cerevisiae strain Ethanol Red, four genes were disrupted in a single transformation with 100% efficiency as well (25). We demonstrated triple-gene knockout in diploid *I. orientalis* was possible, although the disruption efficiency was not as high as in S. cerevisiae. In comparison to other nonconventional yeasts, double- and triple-gene knockouts were achieved in Y. lipolytica with efficiencies of 36.7% and 19.3%, respectively (26). These efficiencies were lower than our reported ones. Furthermore, in a recently published paper, a CRISPR/Cas9-based system was developed in Rhodosporidium toruloides and could achieve single- and double-gene knockouts with efficiencies of 95% and 78%, respectively (27), which are comparable to our single- and double-gene disruption efficiencies. Nevertheless, triple-gene knockout was not shown in *R. toruloides*.

One major concern of the CRISPR/Cas9 technology is the likelihood and unknown consequences of off-target effects (28). However, CRISPR off-target effects could be readily detected in *I. orientalis*. Efficient CRISPR-mediated genome editing in *I. orientalis* relied on the Cas9-induced DSB at the target site and the rescue of DSB lethality by HR. Successful genomic integration of HR repair template then resulted in deletion of the PAM sequence and the last 3 bp of the protospacer, which prevented repeated cleavage by CRISPR/Cas9. If an sgRNA caused nonspecific DSB, DNA repair through HR was unlikely to occur because of the lack of HR disruption donor with homology to the off-target site. Moreover, Cas9 would cause continuous cleavage since the sgRNA target site was not removed. Thus, after transformation of *I. orientalis* with CRISPR/Cas9 plasmid containing both sgRNA and HR disruption donor, if off-target activity happened, very few transformants would survive.

Taken together, we expanded the genetic engineering toolkit for *I. orientalis* with the development of the episomal plasmid and the CRISPR/Cas9-based system. These tools have laid the foundation for CRISPR/Cas9-mediated genome editing in *I. orientalis*. They also serve as the steppingstone that will allow development of complex genome-scale engineering tools, such as CRISPR-AID (8), and further increase the engineering capacity in *I. orientalis*. Future efforts include identification of a CEN sequence and further optimization of our CRISPR/Cas9 system. While ARS-containing plasmid can replicate, it is mitotically unstable and lost at high frequency after each cell division (29). Addition of a CEN to an ARS-containing plasmid helps increase the stability of the plasmid and transmission of the plasmid to daughter cells (30). Other sgRNA expression methods, such as gRNA-tRNA array (31), could potentially increase the multiplexed gene deletion efficiency.

## MATERIALS AND METHODS

### Strains, media, and chemicals.

The strains used in this study are listed in Table S1 in the supplemental material. E. coli transformants were grown at 37°C in LB medium supplemented with 100 μg/ml ampicillin. S. cerevisiae YSG50 and *I. orientalis* SD108 and its mutants were propagated at 30°C in YPAD medium (1% yeast extract, 2% peptone, 0.01% adenine hemisulfate, and 2% dextrose). Yeast transformants were cultured or selected in the Synthetic Complete (SC) dropout medium lacking uracil, tryptophan, leucine, or histidine or with a low concentration of adenine (∼10 mg/liter) (SC-URA, SC-TRP, SC-LEU, SC-HIS, or SC-ADE, respectively). DNA polymerase and restriction enzymes were purchased from New England Biolabs (Ipswich, MA). DNA extraction and purification kits were purchased from Zymo Research (Irvine, CA). All other chemicals were purchased from Sigma (St. Louis, MO) and Fisher Scientific (Pittsburgh, PA). Oligonucleotides, including gBlocks and primers, were all synthesized by Integrated DNA Technologies (IDT; Coralville, IA).

10.1128/mSphere.00345-19.6 TABLE S1 Strains and plasmids used in this study. Download Table S1, DOCX file, 0.01 MB. Copyright © 2019 Tran et al. 2019 Tran et al. This content is distributed under the terms of the Creative Commons Attribution 4.0 International license.

### Plasmid construction.

The plasmid pIo-UG was constructed using the DNA assembler method developed previously (14). In brief, the PCR-amplified fragments, GFP cassette (with TDH3p and Tef1at) and IoURA3 (with URA3p and ENO2t), were cotransformed with ApaI- and NotI-digested pRS415 backbone into S. cerevisiae for *in vivo* assembly via electroporation or lithium acetate-mediated methods. The isolated yeast plasmids were then transformed into E. coli for enrichment, and their identities were verified by restriction digestion or DNA sequencing. The correctly assembled plasmids were subsequently transformed into *I. orientalis* SD108 for target gene expression.

CRISPR/Cas9 plasmids were constructed using DNA assembler from gBlocks containing promoter for sgRNA expression and the following fragments PCR amplified from previous constructs: iCas9, *I. orientalis* URA3 expression cassette, E. coli helper fragment, and S. cerevisiae URA3 expression cassette flanked by XhoI recognition sites and CEN6/ARS4. The resulting plasmids were digested with XhoI to remove S. cerevisiae URA3 expression cassette and religated. The HR donor and spacer sequences were ordered as gBlocks and assembled into CRISPR/Cas9 plasmids by the Golden Gate assembly method (22). Plasmids, key primers, gBlocks, and sequences of RNAP III promoters can be found in the supplemental material (Tables S1 to S4).

10.1128/mSphere.00345-19.7 TABLE S2 List of the main primers used in this study and their sequences. Download Table S2, DOCX file, 0.01 MB. Copyright © 2019 Tran et al. 2019 Tran et al. This content is distributed under the terms of the Creative Commons Attribution 4.0 International license.

10.1128/mSphere.00345-19.8 TABLE S3 List of the main gBlock sequences. Download Table S3, DOCX file, 0.01 MB. Copyright © 2019 Tran et al. 2019 Tran et al. This content is distributed under the terms of the Creative Commons Attribution 4.0 International license.

10.1128/mSphere.00345-19.9 TABLE S4 Sequences of RNA polymerase III promoters and key elements of plasmid. Download Table S4, DOCX file, 0.01 MB. Copyright © 2019 Tran et al. 2019 Tran et al. This content is distributed under the terms of the Creative Commons Attribution 4.0 International license.

### Transformation of *I. orientalis* and its derived mutants.

A fresh 2-ml overnight YPAD culture of *I. orientalis* was diluted to an initial optical density at 600 nm (OD_600_) of 0.2. The cells were continuously grown until they reached an OD_600_ of 0.8 to1. Cells were collected by centrifugation, washed twice with deionized water, and resuspended in 360 μl of transformation mixture containing 240 μl of 50% (wt/vol) polyethylene glycol (PEG) 3350, 36 μl of 1 M lithium acetate, 50 μl of 2-mg/ml DNA from salmon testes (SS-DNA) that was boiled at 100°C for 5 min and quickly chilled on ice, plasmid (1 μg), and deionized water. After mixing thoroughly, the suspension was subjected to heat shock for 1 h at 42°C. Cells were collected by centrifugation and spread on appropriate plates.

### Flow cytometry analysis.

The GFP expression was measured by flow cytometry as described elsewhere (32). Briefly, the transformed *I. orientalis* cells were cultured in SC-URA medium for ∼24 to 120 h and then centrifuged for 2 min at 2,000 × *g* to remove the supernatant. The cell pellets were resuspended in 10 mM phosphate-buffered saline (pH 7.4) and then analyzed by flow cytometry at 488 nm on a BD LSR II flow cytometer analyzer (BD Biosciences, San Jose, CA). After flow cytometry analysis, the *I. orientalis* plasmids were extracted with the Zymoprep Yeast Plasmid Miniprep II kit and retransformed to E. coli for colony counting.

### qPCR.

*I. orientalis* cultures were inoculated from a plate and grown in SC-URA medium until mid-log phase (OD 2 to 3). Total RNA was extracted using the Qiagen RNeasy kit (Venlo, Netherlands), and reverse transcription was performed with the Bio-Rad iScript cDNA synthesis kit (Hercules, CA), with a prior denaturation step at 65°C for 5 min to disrupt gRNA secondary structure. qPCR was performed using Bio-Rad iTaq Universal SYBR green Supermix on a Roche LightCycler 480 qPCR system. *alg9* was used as the reference gene for relative quantification. Experiments were done in biological triplicate.

### Genomic DNA extraction and target site sequencing.

Genomic DNA extraction of *I. orientalis* was performed using methods described elsewhere (33). In brief, 200 μl cell culture was spun down and suspended in 200 μl lithium acetate (200 mM) with 1% SDS. The mixture was incubated at 70°C for 30 min. Then, 600 μl 100% ethanol was added to the mixture. After vortexing, DNA and cell debris were collected by centrifugation and washed with 70% ethanol. The pellet was dissolved in 50 μl water and spun down for 1 min at 13,000 rpm. Two microliters supernatant was used to PCR amplify the target site. The target site was then sequenced by Sanger sequencing (Genewiz, South Plainfield, NJ).

### Calculation of *HIS3*, *LEU2*, or *TRP1*; *SDH1*; double-gene; and triple-gene disruption efficiencies.

Calculations were performed as described in Text S1 in the supplemental material.

10.1128/mSphere.00345-19.1 TEXT S1 Supplemental materials and methods. Download Text S1, DOCX file, 0.01 MB. Copyright © 2019 Tran et al. 2019 Tran et al. This content is distributed under the terms of the Creative Commons Attribution 4.0 International license.
