# Supplementary figures and images for: Development of a CRISPR/Cas9-Based Tool for Gene Deletion in Issatchenkia orientalis
Source: mSphere. 2019 Jun 26;4(3):e00345-19. doi: 10.1128/mSphere.00345-19 (PMC6595149; doi:10.1128/mSphere.00345-19)

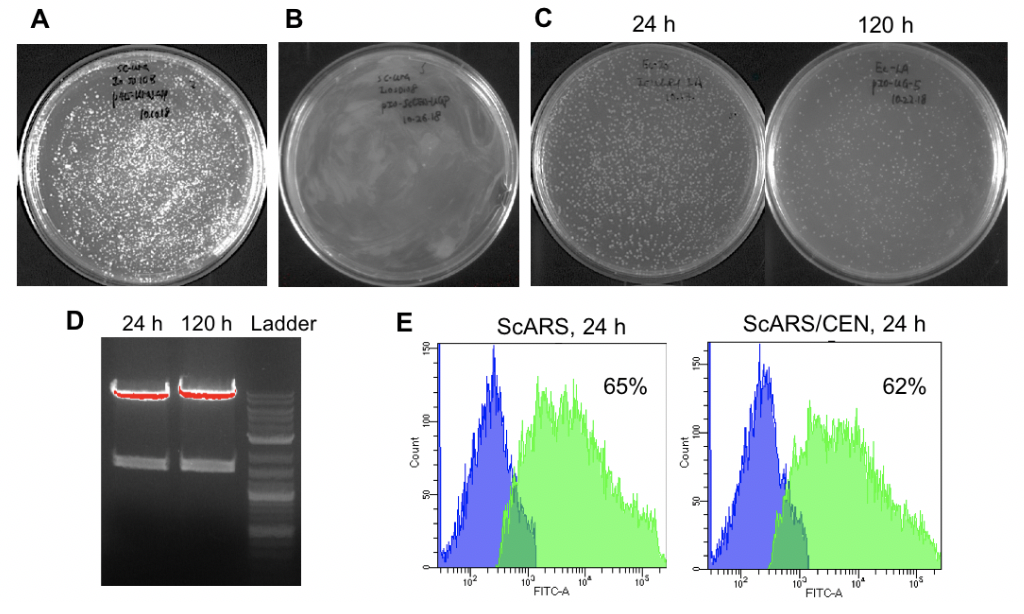

Supplement: FIG S1 [file mSphere.00345-19-sf001.tif]

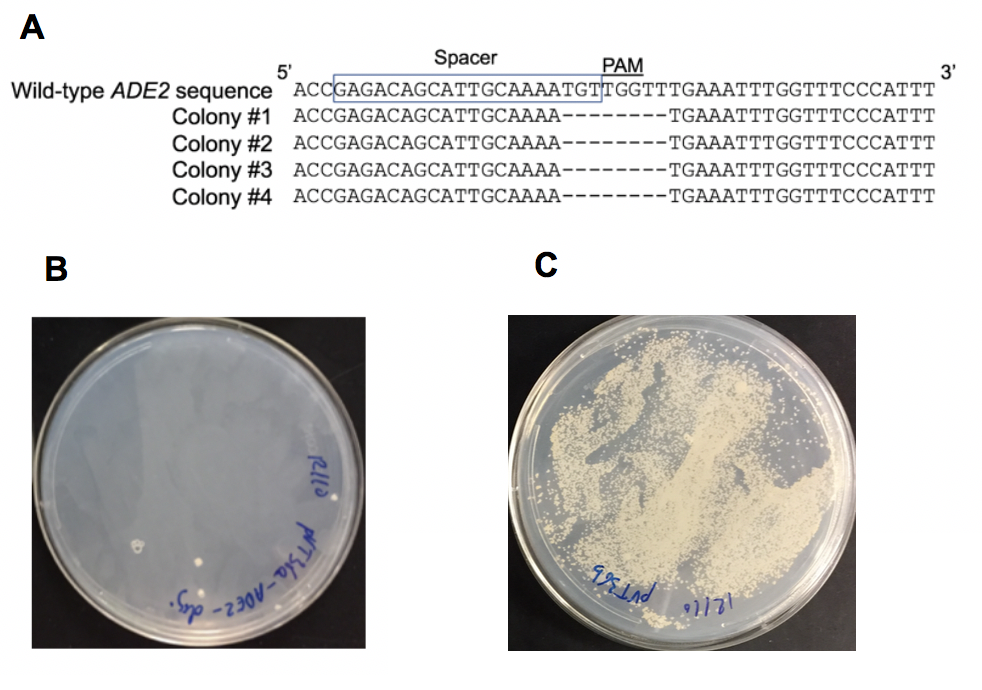

Supplement: FIG S2 [file mSphere.00345-19-sf002.tif]

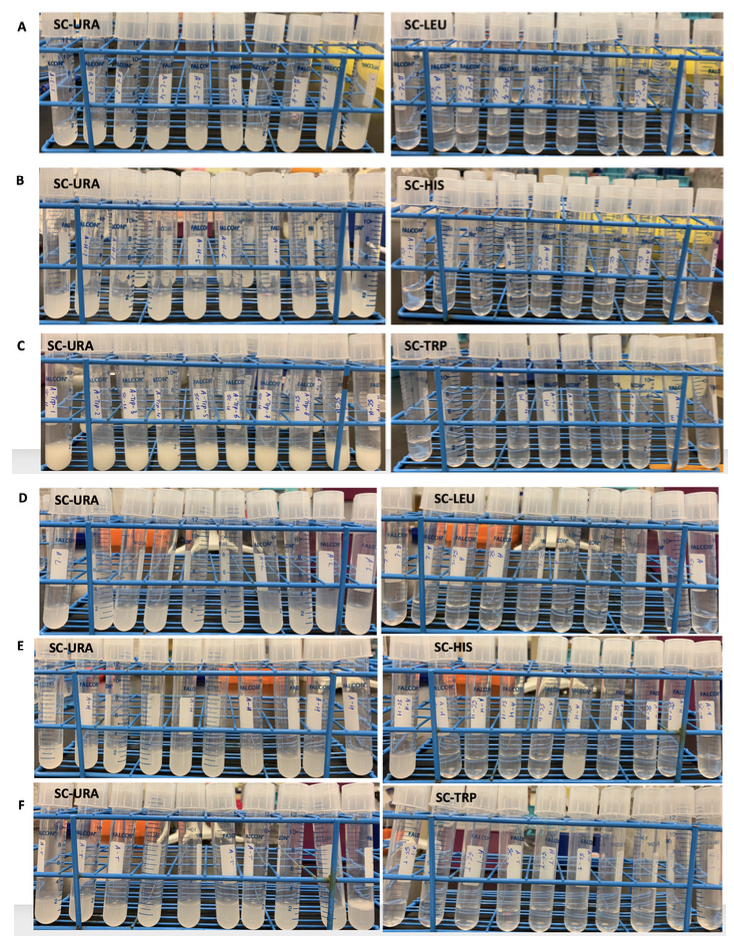

Supplement: FIG S3 [file mSphere.00345-19-sf003.tif]

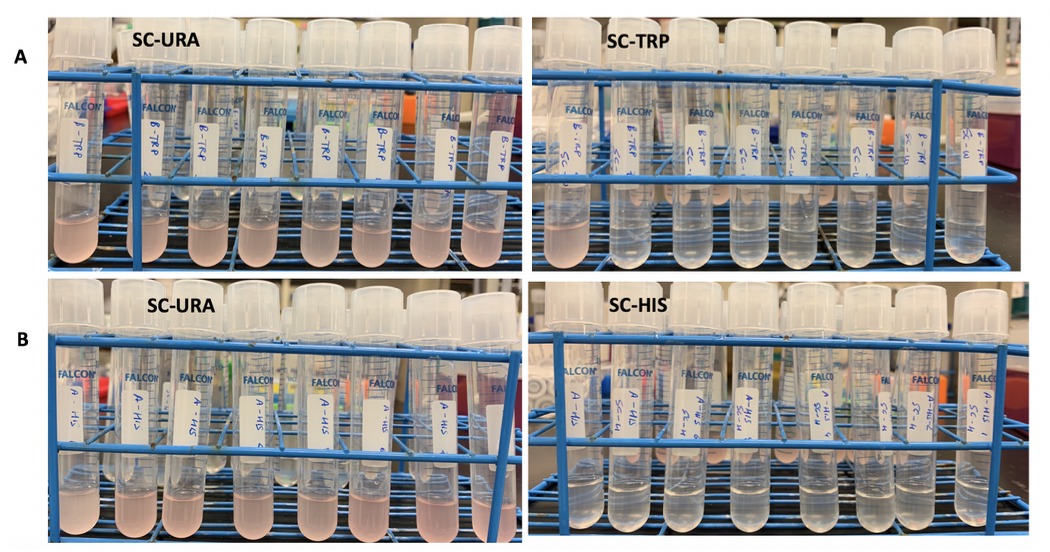

Supplement: FIG S4 [file mSphere.00345-19-sf004.tif]
