# Supplementary material for: Development of a CRISPR/Cas9-Based Tool for Gene Deletion in Issatchenkia orientalis
Source: mSphere. 2019 Jun 26;4(3):e00345-19. doi: 10.1128/mSphere.00345-19 (PMC6595149; doi:10.1128/mSphere.00345-19)
Supplement: TABLE S1 [file mSphere.00345-19-st001.docx]

**Table S1.** Strains and plasmids used in this study.

| **Strains/plasmids** | **Features** | **Sources** |
| --- | --- | --- |
| **Strains** |  |  |
| *I. orientalis* SD108 | *URA3∆*, host for plasmids in this study | Xiao et al. 2014 |
| *S. cerevisiae* YSG50 | *ade2-1, ade3Δ22, ura3-1, his3-11,15, trp1-1, leu2-3,112, can1-100*, used for *in vivo* DNA assembly | Shao et al. 2009 |
| *E. coli* BW25141 | Cloning host | Provided by William Metcalf |
| **Plasmids** |  |  |
| pRS415 | *S. cerevisiae* plasmid containing *LEU2* maker and ARS/CEN | New England Biolabs |
| pIo-UG | Derived from pRS415, containing *E. coli* elements, ScARS, ScLEU2, *IoURA3* and GFP cassette | This study |
| pVT15b-epi | CRISPR/Cas9 plasmid, containing ScARS, *IoURA3*, iCas9, *RPR1* promoter, and sgRNA scaffold | This study |
| pIo-control | Derived from pIo-UG by removing ScARS | This study |
| pVT22-epi | CRISPR/Cas9 plasmid, containing ScARS, *IoURA3*, iCas9, *5S rRNA-tRNA^Leu^* promoter, and sgRNA scaffold | This study |
| pVT24-epi | CRISPR/Cas9 plasmid, containing ScARS, *IoURA3*, iCas9, *5S rRNA* promoter, and sgRNA scaffold | This study |
| pVT25-epi | CRISPR/Cas9 plasmid, containing ScARS, *IoURA3*, iCas9, *tRNA^Ser^* promoter, and sgRNA scaffold | This study |
| pVT36a | CRISPR/Cas9 plasmid, containing ScARS, *IoURA3*, iCas9, *tRNA^Leu^* promoter, and sgRNA scaffold | This study |
| pVT36b | CRISPR/Cas9 plasmid, containing ScARS, *IoURA3*, iCas9, *RPR1’-tRNA^Leu^* promoter, and sgRNA scaffold | This study |
| pVT36b | CRISPR/Cas9 plasmid, containing ScARS, *IoURA3*, iCas9, *RPR1’-tRNA^Leu^* promoter, and sgRNA scaffold | This study |
| pVT36d | CRISPR/Cas9 plasmid, containing ScARS, KanMX marker, iCas9, *RPR1’-tRNA^Leu^* promoter, and sgRNA scaffold | This study |
| pVT36z | CRISPR/Cas9 plasmid, containing ScARS, KanMX marker, iCas9, and 2 *Bsa*I sites | This study |
| pVT36b-X | pVT36b containing spacer sequence targeting *I. orientalis* *ADE2*, *LEU2*, *HIS3*, *TRP1*, or *SDH1* | This study |
| pVT36d-SDH2 | pVT36d containing spacer sequence targeting *I. orientalis* *SDH2* | This study |
| pVT36z-ADE2-HIS3 | pVT36z containing sgRNA expression cassettes for *ADE2* and *HIS3* disruptions | This study |
| pVT36z-ADE2-TRP1 | pVT36z containing sgRNA expression cassettes for *ADE2* and *TRP1* disruptions | This study |
| pVT36z-ADE2-HIS3-SDH2 | pVT36z containing sgRNA expression cassettes for *ADE2*, *HIS3*, and *SDH2* disruptions | This study |
