# Supplementary material for: Development of a CRISPR/Cas9-Based Tool for Gene Deletion in Issatchenkia orientalis
Source: mSphere. 2019 Jun 26;4(3):e00345-19. doi: 10.1128/mSphere.00345-19 (PMC6595149; doi:10.1128/mSphere.00345-19)
Supplement: TABLE S2 [file mSphere.00345-19-st002.docx]

**Table S2.** List of the main primers used in this study and their sequences.

| **Name** | **Sequence (5’→3’)** |
| --- | --- |
| GFP cassette-F | TAACCTAAGGACTTAAATATTTGTACAAACATGTTCCATTGATTTAACCTGATCCAAAAG |
| GFP cassette-R | GGAACAAAAGCTGGAGCTCCACCGCGGTGGCGGCCGCTTGGCTAAAGAATAAGATGAACG |
| IoURA3.cassette.F | GTAATACGACTCACTATAGGGCGAATTGGGTACCGGGCCCGTTGACATTGTCTAGCGGCA |
| IoURA3.cassette.R | TAAAAAATAGACATACCCCTTTTGGATCAGGTTAAATCAATGGAACATGTTTGTACAAAT |
| pVT36.CEN.F | GTTTAAACTTCTTACACAAATCTAGAGCCACCTGGGTCCTTTTC |
| pVT36.CEN.R | AAAAAATGATGAATTGAATTGAACTCGAGGATCGCTTGCCTGTAACTTAC |
| pVT36.URA3Sc.F | CAGGCAAGCGATCCTCGAGTTCAATTCAATTCATCATTTTTTTTTTATTC |
| pVT36.URA3Sc.R | AAAACGCCAGCAACGCGGCCTTTTTCTCGAGCCTGATGCGGTATTTTCTC |
| pVT36.ecoli.F | ACCGCATCAGGCTCGAGAAAAAGGCCGCGTTGCTG |
| pVT36.ecoli.R | CGCGGAACCCCTATTTGTTTATTTTTC |
| pVT36.URA3Io.F | ACGCGTAAACAGGGAAGG |
| pVT36.URA3Io.R | GAAACAGTTTTCATGATGTTTCAAAACCGGTCGTCTGTAGAGTAAAGAAACTTCTCG |
| pVT36.iCas9.F | GTTTCTTTACTCTACAGACGACCGGTTTTGAAACATCATGAAAACTG |
| pVT36.iCas9.R | GAAAAGGACCCAGGTGGCTCTAGATTTGTGTAAGAAGTTTAAAC |
| gRNA.R | TGATTGCCGCTAGACAATGTCAACCTTCCCTGTTTACGCGTCAAAAAAGCACCGACTCGG |
| Ser.gRNA.F | TGAATGTATTTAGAAAAATAAACAAATAGGGGTTCCGCGGGCCACGGTCATTGGCAATTTG |
| 5SRNA.gRNA.F | TGAATGTATTTAGAAAAATAAACAAATAGGGGTTCCGCGGGCGGTTGCGGCCATATCTAC |
| RPR1.gRNA.F | TGAATGTATTTAGAAAAATAAACAAATAGGGGTTCCGCGGGCAGTCCAAAAGGCTCCACC |
| pVT36d.gRNA.R | CTTTTGGATCAGGTTAAATCAAAAAAAAGCACCGACTCGG |
| pVT36d.KanMX.F | GCACCGAGTCGGTGCTTTTTTTTGATTTAACCTGATCCAAAAG |
| pVT36d.KanMX.R | ATTTTACATTCAGATGTCATTAGAAAAACTCATCGAGC |
| pVT36d.PDC1t.F | CATTTGATGCTCGATGAGTTTTTCTAATGACATCTGAATGTAAAATGAAC |
| pVT36d.PDC1t.R | TATGCTTCACAGAGGGTG |
| gRNA-1.F | CTTTGGTCTCCTGCATAAACAAATAGGGGTTCC |
| gRNA-1.R | GAAAGGTCTCCGGCCTCAACCTTCCCTGTTTAC |
| gRNA-2.F | CTTTGGTCTCCGGCCTAAACAAATAGGGGTTCC |
| gRNA-2.R | GAAAGGTCTCCAAACTCAACCTTCCCTGTTTAC |
| gRNA-3.F | CTTTGGTCTCCTCGGTAAACAAATAGGGGTTCC |
| gRNA-3.R | GAAAGGTCTCCCCGATCAACCTTCCCTGTTTAC |
| qPCR.ADE2.F | GAGACAGCATTGCAAAATG |
| qPCR.ADE2.R | ACTCGGTGCCACTTTTTC |
| alg9.ref.F | GCTGCGCTACGTTCTATATG |
| alg9.ref.R | CCCAAGTCTGCTTACCAAAG |
| ADE2.seq.F | ﻿TGAACACATTGATGGTTCATTC |
| ADE2.seq.R | ﻿TCTTTTACAACATAGTTACCTCTAC |
| HIS3.seq.F | ATGTCTCCACAGAAGAAGC |
| HIS3.seq.R | CTCTTTTCAAACCTAGTTCTA |
| LEU2.seq.F | TGTCTACCAAAAATATCGTTC |
| LEU2.seq.R | TTGAGCTTCGTTGGAGACT |
| TRP1.seq.F | ﻿ATGGCAAAGATTATCAAAG |
| TRP1.seq.R | TCGGAATCTAATAATGGAATG |
| SDH1.seq.F | ﻿ATGCTCTCTCTACAAAGAAG |
| SDH1.seq.R | ﻿ACAACTTGGAAATACATGC |
| SDH2.seq.F | ﻿AAAGATTAGCAGAACCAG |
| SDH2.seq.R | ﻿GTTTCTTGGTCTGTACTTG |
