# Supplementary material for: Development of a CRISPR/Cas9-Based Tool for Gene Deletion in Issatchenkia orientalis
Source: mSphere. 2019 Jun 26;4(3):e00345-19. doi: 10.1128/mSphere.00345-19 (PMC6595149; doi:10.1128/mSphere.00345-19)
Supplement: TABLE S3 [file mSphere.00345-19-st003.docx]

**Table S3.** List of the main gBlock sequences.

| **Name** | **Sequence (5’→3’)** |
| --- | --- |
| pVT36.Leu.gRNA | CATATTTGAATGTATTTAGAAAAATAAACAAATAGGGGTTCCGCGGGCGCCCTCAATGACAGCATAAGTGGCCGAGCGGTCTAAGGCGACAGACTCAAGTTCTGTTACTCGTAAGAGTGCGCAGGTTCGAACCCTGTCTTATGCAGGAGACCGAGGTCTCCGTTTTAGAGCTAGAAATAGCAAGTTAAAATAAGGCTAGTCCGTTATCAACTTGAAAAAGTGGCACCGAGTCGGTGCTTTTTTCCTAGGACGCGTAAACAGGGAAGGTTGACATTGTCTAGCGGCAATCATTGT |
| ADE2 | CTTTGGTCTCCTGCAGAATTCGCAGTTGCAGACTCTGTTAGCGTTGAAAGCACCGAGACAGCATTGCAAAATGAAATTTGGTTTCCCATTTATGCTGAAGTCCAAAACTGAAGCATATGATGAGACAGCATTGCAAAATGTGTTTGGAGACCTTTC |
| ADE2  (for *RPR1*) | CTTTGGTCTCCCGGTGAATTCGCAGTTGCAGACTCTGTTAGCGTTGAAAGCACCGAGACAGCATTGCAAAATGAAATTTGGTTTCCCATTTATGCTGAAGTCCAAAACTGAAGCATATGATGAGACAGCATTGCAAAATGTGTTTGGAGACCTTTC |
| ADE2  (for *tRNA^Ser^*) | CTTTGGTCTCCGTCGGAATTCGCAGTTGCAGACTCTGTTAGCGTTGAAAGCACCGAGACAGCATTGCAAAATGAAATTTGGTTTCCCATTTATGCTGAAGTCCAAAACTGAAGCATATGATGAGACAGCATTGCAAAATGTGTTTGGAGACCTTTC |
| LEU2 | CTTTGGTCTCCTGCAGAATTCGGACATTTTGATTGTTAGAGAGTTGGTTGGAGGTATCTACTTTGGGGAGAGACACGGGAAGTGGTAGAGCGTGGGATACTGAGGCGTACTCGGTTGAGGATATCTACTTTGGGGAGAGAGGTTTGGAGACCTTTC |
| HIS3 | CTTTGGTCTCCTGCAGAATTCCTGGAATTGGGTTTTTGGATCATATGCTACACGCATTAGCCAAACATTCACCTCATTGTTGAGTGCATTGGTGATTTACATATTGACGACCATCACACATCATTAGCCAAACATTCAGGGGTTTGGAGACCTTTC |
| TRP1 | CTTTGGTCTCCTGCAGAATTCGAACGGTTGATCCTCAACAAGCCATCGAAATATCCAAGCTATGTCGAGCACTCAAACGGGTCCAGATTTGTGGATTCAAGAGATTTACTAAATTATATGACCAAGCTATGTCGAGCAAAGGTTTGGAGACCTTTC |
| SDH1-spacer | CTTTGGTCTCATGCAATAATCCTTTGCCATTGCGGGTTTTAGAGCTAGAAATAGCAAGTTAAAATAAGGCTAGTCCGTTATCAACTTGAAAAAGTGGCACCGAGTCGGTGCTTTTTTTCTAGAGGCCAGAGACCTTTC |
| SDH1-HR | CTTTGGTCTCAGGCCGAATTCAACAACACAGTCGTATTCATGGTCAATGACATGATAATCCTTTGCCATTGGCCTCTTTACCATTTAGGTTGTTAAATCTGTTGATGACTTGCCTTGAGGCGTTTAGAGACCTTTC |
| SDH2-spacer | CTTTGGTCTCATGCAGCTGAAGGTGAATCCAGTGGGTTTTAGAGCTAGAAATAGCAAGTTAAAATAAGGCTAGTCCGTTATCAACTTGAAAAAGTGGCACCGAGTCGGTGCTTTTTTTCTAGAGGCCAGAGACCTTTC |
| SDH2-HR | CTTTGGTCTCAGGCCGAATTCGCGGATCAGGCACCAGACGTGGCTGTACCGGCAGCTGAAGGTGAATCCAGGTTGTACGACAGGTGAGATGGGGGAAGCCGGGCCCAAGTAAGAGGCTCCTGTTTAGAGACCTTTC |
