# Supplementary material for: Development of a CRISPR/Cas9-Based Tool for Gene Deletion in Issatchenkia orientalis
Source: mSphere. 2019 Jun 26;4(3):e00345-19. doi: 10.1128/mSphere.00345-19 (PMC6595149; doi:10.1128/mSphere.00345-19)
Supplement: TEXT S1 [file mSphere.00345-19-s0001.docx]

**Method S1.** Supplemental materials and methods

**Calculation of *HIS3, LEU2, or TRP1* disruption efficiencies.** *I. orientalis* cells were transformed with CRISPR/Cas9 plasmids targeting *HIS3*, *LEU2*, *or TRP1* genes. For each knockout, 10 colonies were inoculated in 2 mL of SC-URA. 50 µL of cell culture were transferred into 2 mL of fresh SC-URA media every 24 hours for 2 days. Then, 50 µL of cell culture were transferred into 2 mL of SC-URA and 2 mL of SC minus the appropriate compound produced by the target gene, SC-HIS (SC-histidine), SC-LEU (SC-leucine), or SC-TRP (SC-tryptophan). Cells were allowed to grow for 2 days, and *HIS3*, *LEU2*, or *TRP1* disruption efficiencies were calculated as the proportion of colonies that grew in SC-URA but not in SC-HIS, SC-LEU, or SC-TRP, respectively. Experiments were done in biological duplicate.

**Calculation of *SDH1* disruption efficiency.** *I. orientalis* cells were transformed with CRISPR/Cas9 plasmid targeting *SDH1* gene. Following transformation, 8 randomly picked colonies were inoculated in 2 mL of YPAD and grown until saturation. Genomic DNA was then extracted, and *SDH1* target site was amplified by PCR and sent for sequencing. *SDH1* disruption efficiency was calculated as the percentage of disrupted colonies.

**Calculation of double gene disruption efficiency.** *I. orientalis* cells were transformed with CRISPR/Cas9 plasmids targeting the *ADE2* and *HIS3*, or *ADE2* and *LEU2* genes. All cells were plated on SC-URA after transformation and incubated in 30 °C. *ADE2* disruption efficiency was determined as the proportion of red colonies on the plate. For each knockout, 8 red colonies were randomly picked and inoculated in SC-URA. 50 µL of cell culture were subcultured into 2 mL of fresh SC-URA media every 24 hours for 2 days. Then, 50 µL of cell culture were transferred into 2 mL of SC-URA and 2 mL of SC-HIS or SC-LEU, depending on the knockout. Cells were allowed to grow for 2 days, and *HIS3* and *LEU2* disruption efficiencies were calculated as the proportion of colonies that grew in SC-URA but not in SC-HIS or SC-LEU, respectively. The overall double gene knockout efficiency was estimated as the *ADE2* disruption efficiency multiplied by *HIS3* or *LEU2* disruption efficiency. Experiments were done in biological duplicate.

**Calculation of triple gene disruption efficiency.** *I. orientalis* cells were transformed with CRISPR/Cas9 plasmid targeting the *ADE2*, *HIS3*, and *SDH2* genes. All cells were plated on SC-URA after transformation and incubated in 30 °C. *ADE2* disruption efficiency was calculated as the percentage of red colonies on the plate. 8 randomly selected red colonies were inoculated in SC-URA. 50 µL of cell culture were transferred into 2 mL of fresh SC-URA media every 24 hours for 2 days. Genomic DNA was then extracted, and the *HIS3* and *SDH2* target sites were PCR-amplified and sent for sequencing. Triple gene disruption efficiency was determined as the overall *ADE2* disruption efficiency multiplied by the proportion of screened colonies with both *HIS3* and *SDH2* disrupted.
